# Targeting MEN1-deficient tumors with DHODH inhibitor

**DOI:** 10.1016/j.jncc.2022.03.001

**Published:** 2022-04-06

**Authors:** Lei Zheng

**Affiliations:** 1 Department of Oncology, Johns Hopkins University School of Medicine, Baltimore, MD, USA; 2 Multidisciplinary Gastrointestinal Cancer Laboratories Program, the Sidney Kimmel Comprehensive Cancer Center, Johns Hopkins University School of Medicine, Baltimore, MD, USA; 3 Department of Surgery, Johns Hopkins University School of Medicine, Baltimore, MD, USA; 4 Pancreatic Cancer Precision Medicine Center of Excellence Program, Johns Hopkins University School of Medicine, Baltimore, MD, USA

The ultimate goal of cancer genetics is to exploit molecular changes in a cancer to design tumor specific therapies. *Ma et al.* in their recent paper in *Cell Research* used an elegant strategy, a CRISPR-Cas9 synthetic lethal knockout screen, to identify vulnerabilities in MEN1 deficient tumor cells in cell culture. They then successfully translated these results into the development of candidate gene targets for possible drug therapy.

MEN1 deficiency occurs in sporadic neuroendocrine tumors, and germline mutations in the gene are the basis for cancer predisposition in the human multiple endocrine neoplasia (MEN1) syndrome^1^^,^^2^. Such patients are at high risk for the development of a variety of neuroendocrine neoplasms, including pancreatic neuroendocrine carcinomas, which are often not amenable for curative surgical resection and account for the mortality of MEN1 patients^3^.

The *in vitro* genome-wide screen in U251 *MEN1*-/- cells yielded two candidate genes required for viability, carbamoyl-phosphate synthetase 2, aspartate transcarbamylase, and dihydroorotase (CAD) and dihydroorotate dehydrogenase (DHODH)^4^^,^^5^. Both are rate-limiting enzymes involved in pyrimidine biosynthesis. Deletion of either gene significantly reduced viability of *MEN1* deficient but not *MEN1* wild type U251 cells.

Leflunomide, a drug in use for the treatment of rheumatoid arthritis, targets one of these genes, DHODH^6^. The *MEN1*-/- U251 cells in culture were four-fold more sensitive to leflunomide than *MEN1* wild type U251 cells. *In vivo*, leflunomide treatment delayed tumor growth of *MEN1*-/- cells implanted in nude mice. However, this anti-tumor effect of leflunomide could be reversed by increasing the concentration of pyrimidine metabolites, orotate and uridine, indicating that *MEN1*-/- cells require increased levels of critical pyrimidine intermediates for survival.

Because leflunomide has been in clinic for over 30 years, the authors attempted to translate their laboratory findings and repurpose the drug for the treatment of MEN1 syndrome patients in a clinical trial. In this clinical trial of patients who were diagnosed with advanced pancreatic neuroendocrine tumors and whose tumors had become refractory to the standard of care treatment, all three participants exhibited stable disease following the treatment with leflunomide for more than 11 months. Importantly, no participants experienced adverse effects during the leflunomide treatment.

Finally, the authors asked whether leflunomide might prevent the development of tumors in the context of germline deficiency of MEN1. In the *Men*+/- mouse model of MEN1 syndrome, the frequency of spontaneous tumor development decreased from 65% to 5% in *Men*+/- mice that received continuous leflunomide treatments starting at 6 months old. These results thus support testing leflunomide as a potential breakthrough chemopreventive treatment for those individuals with germline mutations in *MEN1*.

In summary, Ma et al. have presented a potential therapy option for the treatment of sporadic and germline *MEN1*-mutated pancreatic neuroendocrine tumors. Their study provided a successful example of utilizing the synthetic lethal genome-wide screening to identify novel therapeutics for neoplasms caused by a known genetic alteration.

## Declaration of competing interest

L.Z. receives grant support from Bristol-Myers Squibb, Merck, Astrazeneca, iTeos, Amgen, NovaRock, Inxmed, and Halozyme. L.Z. is a paid consultant/Advisory Board Member at Biosion, Alphamab, NovaRock, Ambrx, Akrevia/Xilio, QED, Natera, Tempus, Pfizer, Johnson and Johnson, Novagenesis, Snow Lake Capitals, BioArdis, Amberstone, and Mingruizhiyao. L.Z. holds shares at Alphamab and Mingruizhiyao.
